# Effect of Mixed Probiotics on Alleviating H1N1 Influenza Infection and Regulating Gut Microbiota

**DOI:** 10.3390/foods13193079

**Published:** 2024-09-27

**Authors:** Hongchao Wang, Yuhao Zhao, Zhangming Pei, Jianxin Zhao, Pinghu Zhang, Xinyue Zhang, Zhijian Zhang, Wenwei Lu

**Affiliations:** 1 State Key Laboratory of Food Science and Resources, Jiangnan University, Wuxi 214122, China; hcwang@jiangnan.edu.cn (H.W.); jxzhao@jiangnan.edu.cn (J.Z.); 2 School of Food Science and Technology, Jiangnan University, Wuxi 214122, China; 3 Jiangsu Key Laboratory of Integrated Traditional Chinese and Western Medicine for Prevention and Treatment of Senile Diseases, Medical College, Yangzhou University, Yangzhou 225009, China; zhangpinghu@163.com (P.Z.);; 4 Department of Nephrology, The Affiliated Wuxi People’s Hospital of Nanjing Medical University, Wuxi People’s Hospital, Wuxi Medical Center, Nanjing Medical University, 299 Qingyang Rd, Wuxi 214000, China; 5 National Engineering Research Center for Functional Food, Jiangnan University, Wuxi 214122, China

**Keywords:** influenza virus, gut microbiota, antiviral probiotic, systemic immunity, *Lactobacillus*

## Abstract

Influenza and other respiratory infections cause annual epidemics worldwide, with high incidence and mortality rates reported among immunocompromised infants and elderly individuals. Probiotics can modulate the immune system through their bacterial compositions and metabolites, affecting influenza infections and effectively responding to viral mutations. Therefore, we evaluated the anti-influenza effects of mixed probiotics administered orally before and after influenza infection. The results showed that the mixed probiotics consisting of *Lacticaseibacillus rhamnosus* CCFM1279, *Limosilactobacillus reuteri* CCFM1145, and *Lacticaseibacillus casei* CCFM1127 inhibited viral replication and reduced lung inflammatory damage against influenza. In addition, the mixed-probiotics treatment activated the systemic immune response of the host. The gut microbiota analysis revealed a notable increase in the abundance of *Alistipes* and *Rikenella* following mixed-probiotic supplementation. The metabolomic analysis indicated a significant increase in adenosine levels, which was positively correlated with the abundance of *Parvibacter*. These findings highlight the effectiveness of mixed probiotics in fighting influenza viruses and suggest that certain gut microbiota and their metabolites may play a significant role in influencing the outcomes of influenza infections.

## 1. Introduction

Influenza is a prevalent and highly contagious respiratory disease known for causing substantial lung inflammation [1,2]. Additionally, it can inflict damage to the intestine to some extent [3]. Its impact should not be underestimated. Every year, the influenza virus sweeps across the globe, imposing a significant burden on public health and society [4]. Despite the widespread adoption of vaccination as an effective preventive measure, the current vaccine system is struggling to rapidly and adaptively counter the challenges posed by the influenza virus [5].

The gut environment plays a crucial role in regulating immune system function. Extensive evidence indicates a close link between the intestinal microbiota, their metabolites, and lung health [6]. It has been shown that elevated levels of short-chain fatty acids in the gut can stimulate the expansion of lung dendritic cell precursors, thereby playing a crucial role in shaping the pulmonary immune environment [7]. Furthermore, the intestinal immune barrier, composed of immunoglobulin A, mucosal lymphoid cells, and mesenteric lymph nodes, forms a critical defense system against exogenous pathogens [8]. Therefore, researchers focused on the role of the gut–lung axis, which refers to the mutual influence of the intestine and lungs. The gut microbiota, the microbial community residing within the intestines, is also believed to play a pivotal role in maintaining immune homeostasis in the body. Accumulating evidence also suggests a close association between the gut microbiota and respiratory health. It has been shown that an imbalance in the gut microbiota may increase the risk of respiratory diseases such as influenza through its interactions with the immune system [9].

Although research has shown that specific probiotics can modulate the immune system through the gut microbiota, thereby influencing respiratory immune responses or enhancing vaccine efficacy to protect against influenza virus infections [10,11,12], probiotics have not been widely used in the context of influenza virus infection. As global competition intensifies in the search for more potent strains, it becomes increasingly essential to conduct carefully designed in vivo studies to validate the efficacy of promising probiotics. Previously, researchers combined a strain of *Lactobacillus plantarum* with anti-influenza properties, along with a strain of *L. rhamnosus* known for modulating immune functions [13] and a strain of *L. paracasei* known for its protective effects on intestinal barriers, to form a mixed probiotic. Therefore, this study aimed to evaluate influenza symptoms from multiple perspectives and to validate the effectiveness of probiotics in animal models.

## 2. Materials and Methods

### 2.1. Bacterial Strains and Culture Conditions

*Lacticaseibacillus rhamnosus* CCFM1279, *Limosilactobacillus reuteri* CCFM1145, and *Lacticaseibacillus casei* CCFM1127 were obtained from the Culture Collection of Food Microbiology at Jiangnan University. These probiotics were cultured in De Man, Rogosa, and Sharpe-medium supplemented with 0.05% (*w*/*v*) L-cysteine-HCl at 37 °C for 12 h under anaerobic conditions using an AW500SG system (Electrotek Scientific Ltd., Keighley, UK).

### 2.2. Treatment of Mice In Vivo

All in vivo experiments were conducted according to the guidelines approved by the Ethics Committee of Yangzhou University (Approval No. 202306835). The animals used in this study were 3–4 week-old female ICR mice purchased from the Comparative Animal Medicine Center of Yangzhou University (Yangzhou, Jiangsu, China). These mice were fed a conventional rodent diet and provided unrestricted access to water in controlled environments: a 12-h light/dark cycle, a temperature of 25 ± 2 °C, and humidity of 50%.

After 10 days of adaptation, the mice were randomly divided into four groups (n = 10 each group). Starting from the 10th day, the mice received a daily gavage of either 200 µL of bacterial suspension (1.05 × 10^10^ CFU per mouse per day) or an equivalent volume of saline solution.

On the 17th day, the mice were infected with the influenza A virus A/FM1/47 (H1N1) via nasal inoculation at a 50% lethal dose (LD50) provided by the Key Laboratory of Avian Infectious Diseases of Yangzhou University. The mice in the treatment group were administered oseltamivir immediately after infection. The mice in the Mix group continued to receive daily gavage with bacterial suspension after being infected with the influenza virus, while the other groups received an equal amount of saline solution by gavage until they were sacrificed on the 22nd day. All mice were weighed daily following influenza virus H1N1 infection.

### 2.3. Lung Tissue Cellular Injury

After euthanizing the mice, we performed three lung washings using 800 µL phosphate buffered saline, all of which was extracted and centrifuged at 1500× *g* for 15 min at 4 °C to obtain the bronchoalveolar lavage fluid (BALF). To assess the cytotoxicity in the lungs, we measured lactate dehydrogenase (LDH) activity in the BALF using an LDH activity assay kit (Solarbio Life Sciences Co., Ltd., Beijing, China). The number of biological replicates was five.

### 2.4. Histopathological Analysis of Lung Tissue

The left lung lobes were preserved in formalin prior to fixation. Following embedding in paraffin blocks, the lung tissues were sectioned into 5-µm-thick slices and stained with hematoxylin and eosin. The tissue slices were photographed using a Pannoramic MlDI digital scanner (3DHistech Ltd., Budapest, Hungary). The number of biological replicates was 10.

### 2.5. Viral Load Quantification and mRNA Expression Analysis of Antiviral Proteins

After sacrificing the mice, their lungs were dissected and placed in RNase-free tubes, immersed in liquid nitrogen, and subsequently preserved at a temperature of −80 °C. The lung tissue samples were processed to extract the total RNA, utilizing the Trizol reagent (Thermo Fisher Scientific Co., Ltd., Waltham, MA, USA). Subsequently, this RNA was converted into complementary DNA (cDNA) with the aid of the HiScript^®^ II SuperMix (Vazyme Biotech Co., Ltd., Nanjing, China). After mixing the cDNA with a ChamQ Universal SYBR qPCR Master Mix (Vazyme Biotech Co., Ltd., Nanjing, China) according to the guidelines provided by the manufacturer, a quantitative PCR was conducted using a CFX96 Thermal Cycler (Bio-Rad Laboratories, Hercules, CA, USA). The gene expression was quantified by the 2^−ΔΔCT^ method, with the results standardized relative to the expression of the housekeeping gene GAPDH. The sequences of the primers used are detailed in Table 1. The number of biological replicates was five.

### 2.6. Cytokine Quantification

The serum was frozen at −20 °C. The TNF-α, interleukin (IL)-6, IL-10, and IL-1β levels were measured using commercial ELISA kits (R&D Systems, Minneapolis, MN, USA). The number of biological replicates was five.

### 2.7. Blood Cell Analysis

The mice blood was collected at room temperature in anticoagulant tubes containing EDTA, ensuring thorough contact with the EDTA. The lymphocyte, monocyte, neutrophil, and eosinophil counts were analyzed using an Automated Hematology Analyzer. The number of biological replicates was five.

### 2.8. Gut Microbiota Analysis

Fifty milligrams of freeze-dried stool were weighed. Adhering to the protocol outlined by the manufacturer, an MP kit (MP Biomedicals, Santa Ana, CA, USA) was used for the extraction of the total DNA from the stool sample. The amplification of the 16S rRNA gene was carried out using the V3-V4 primer set, which included the forward primer 341F (sequence: 5′-CCTAYGGGRBGCASCAG-3′) and the reverse primer 806R (sequence: 5′-GGACTACNVGGGTATCTAAT-3′). The PCR process began with a pre-denaturation phase at a temperature of 95 °C, lasting for 5 min. This was succeeded by 30 cycles of the PCR amplification phase, which included denaturing at 95 °C for 30 s, annealing at 52 °C for 30 s, and extension at 72 °C for another 30 s. Concluding the PCR, a final extension was executed at 72 °C for a duration of 7 min. The PCR products were then separated and retrieved using agarose gel electrophoresis, and a Qiagen QiaQuick PCR Purification Kit (Qiagen, Hilden, Germany) was used to purify the amplification products. Afterward, the purified DNA samples were sent for sequencing, utilizing the Illumina MiSeq system at Jiangnan University. The sequencing data were then subjected to quality control using the QIIME2 2019.7 software, with a quality score threshold set at q < 20. After obtaining an operational taxonomic unit table, community diversity and intergroup microbial community differentiation analyses were conducted. The number of biological replicates was five.

### 2.9. Fecal Metabolomics

The fecal metabolites were examined through the application of liquid chromatography–mass spectrometry (LC-MS) technology (Thermo Fisher Scientific, Waltham, CA, USA). Briefly, the fecal samples were first mixed thoroughly with 200 μL of double-distilled water (ddH_2_O). Subsequently, they were blended with 800 microliters of methanol at a ratio of one part water to one part methanol. After incubating the mixture at −20 °C for 1 h, the proteins were precipitated via centrifugation at 15,000× *g* for 15 min at 4 °C. The liquid layer above was subsequently evaporated to dryness. Subsequently, the dried sample was dissolved in 200 μL of a methanol solution (methanol:water, 1:1) and centrifuged (15,000× *g* for 15 min at 4 °C) to collect the supernatant. Post-filtration through a 0.22-μm membrane, the sample underwent an LC-MS analysis under the column and condition settings that were previously described by Zhu [14]. The number of biological replicates was five.

### 2.10. Statistic Analysis

Using Origin 2021 and IBM SPSS Statistics 26.0 software for statistical analysis, the data were expressed as the mean ± standard error of the mean (SEM). Student’s *t*-test was employed to assess statistical differences between two groups, while ANOVA was used to evaluate statistical differences among multiple groups. * *p* < 0.05, ** *p* < 0.01, **** *p* < 0.0001 was considered statistically significant.

## 3. Results

### 3.1. A Mixed Probiotics Solution Protects Mice from Influenza Virus Infection

**Figure 1:**
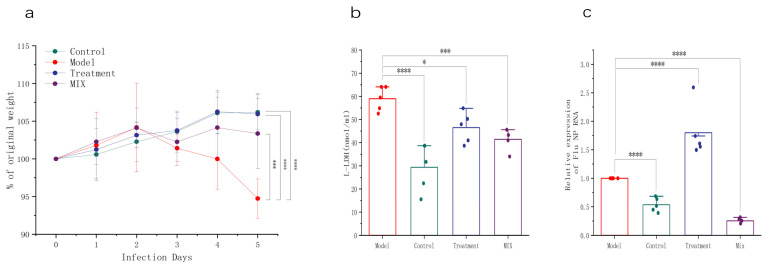
Mixed probiotics protect mice against influenza. (**a**) Percentage of daily weight loss following influenza infection (*n* = 10). (**b**) Total LDH levels measured in the BALF (*n* = 5). (**c**) Amount of viral RNA in the lung tissues (*n* = 5) as indicated by *NP* gene expression. * *p* < 0.05, *** *p* < 0.001, **** *p* < 0.0001.

To investigate the alleviative effect of mixed probiotics on influenza infection, the mice were continuously orally administered mixed probiotics before and after H1N1 infection. The control and treatment groups that were administered oseltamivir via gavage showed a trend toward weight gain during the intervention period. On the third day post-H1N1 infection, the mice in the Model group began to experience weight loss, which persisted until they were sacrificed. By the fifth day post-infection, the average weight of the mice in the Model group had significantly decreased to 94.93% of that in the Control group. In contrast, the mice in the mixed probiotic (Mix) group showed a mitigating trend in weight loss on the fourth day post-infection, indicating that the oral administration of mixed probiotics significantly alleviated weight loss in the mice during the late stage of influenza infection (Figure 1a).

The detection of the influenza virus NP RNA in mice lung tissues can indicate the replication status of the pathogen in vivo. Compared to the model group, the expression level of NP RNA in the lungs of the Control group mice was significantly reduced. The mice in the Mix group, after the oral administration of the mixed probiotic, showed a significant inhibition of viral replication in the lungs, with NP RNA expression levels returning to those of the Control group. (Figure 1c). Additionally, as expected, lung lactate dehydrogenase (L-LDH) activity in the uninfected Control mice was lower than in the Model group, indicating minimal lung cell damage and improved lung integrity. Compared with the Model group, the oral administration of mixed probiotics significantly reduced L-LDH activity, alleviating H1N1-induced lung damage (Figure 1b).

### 3.2. Impact of Mixed Probiotics on the Systemic Immune Response in Mice

**Figure 2:**
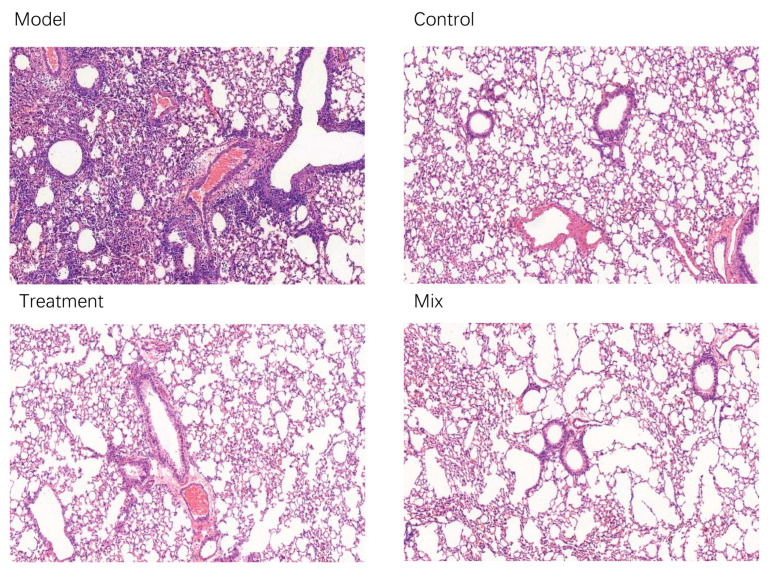
Representative micrographs of the lung tissue samples stained with hematoxylin and eosin (*n* = 10). Magnification: 400×.

Lung histopathology is a critical indicator of the severity of respiratory infection. Following H1N1 infection, the activation of the host’s immune response occurs in the lung cells, leading to the release of inflammatory factors. The immune cells are then activated and aggregate in the infected area, resulting in localized inflammation and cellular infiltration, which clears the H1N1 virus and prevents further deterioration. In the Control group, smooth and intact bronchi without any signs of inflammation or bleeding were observed. In contrast, the Model group exhibited severe inflammatory cell infiltration, bronchial rupture, incomplete mucosa, and localized bleeding (Figure 2). The oral administration of mixed probiotics significantly alleviated lung pathology in the mice, with no infiltration of the inflammatory cells around the bronchi and blood vessels and relatively intact alveolar structures, similar to the findings in the Control group (Figure 2).

**Figure 3:**
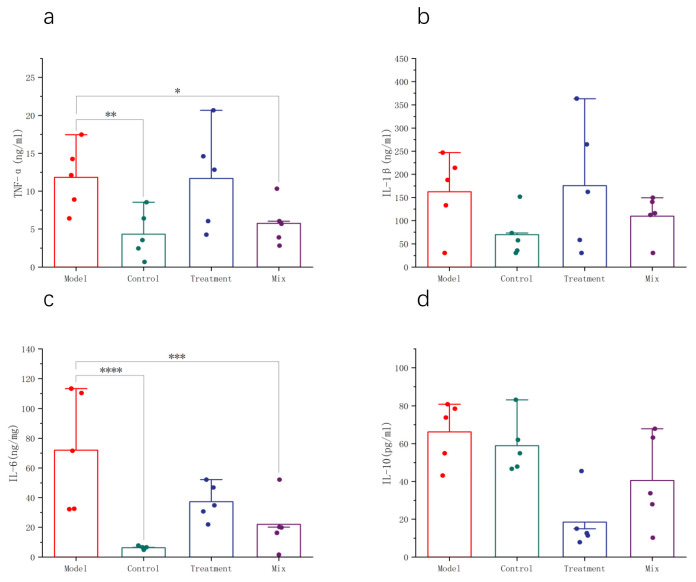
Administration of mixed probiotics regulates the host’s systemic immunity. Shown are the levels of (**a**) TNF-α, (**b**) IL-1β, (**c**) IL-6, and (**d**) IL-10 in the serum (*n* = 5). * *p* < 0.05, ** *p* < 0.01, *** *p* < 0.001,**** *p* < 0.0001.

Serum inflammatory cytokine levels reflect the degree of immune system activation, the intensity of inflammatory responses, and the effectiveness of cellular immunity during influenza infection. Following the influenza virus infection in the mice, significant increases in IL-6 and TNF-α concentrations in the serum indicated the activation of the immune system and the initiation of inflammatory responses to combat viral infection. However, elevated concentrations of inflammatory cytokines may lead to excessive inflammation, tissue damage, and the development of complications. Following the oral intake of a blend of probiotics, there was a marked decrease in the serum levels of the pro-inflammatory cytokines IL-6 and TNF-α. This reduction in IL-6 levels was greater than that induced by oseltamivir, suggesting that the treatment may inhibit the excessive release of pro-inflammatory cytokines, thereby alleviating the systemic inflammatory response (Figure 3a,c). IL-10, an anti-inflammatory cytokine, helps maintain the immune response balance and mitigates tissue damage caused by excessive inflammation. In comparison to the Model group, the serum IL-10 levels remained essentially unchanged in the Control and the Mix groups, potentially playing a role in maintaining immune system equilibrium during the influenza infection (Figure 3d). The serum IL-1β concentrations did not show significant changes after the influenza infection. Considering the dynamic changes in the inflammatory cytokines during the influenza infection, this may be related to sampling timing, warranting further investigation (Figure 3b).

**Figure 4:**
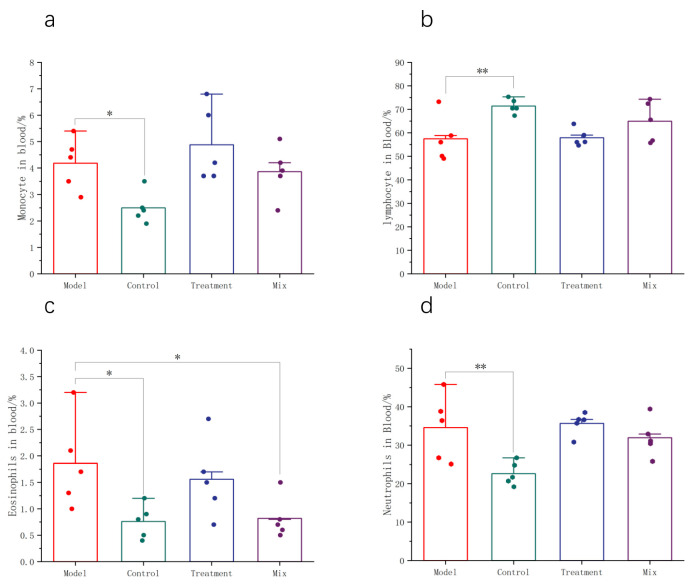
Administration of mixed probiotics regulates the host’s immune cells. Levels of (**a**) monocytes, (**b**) lymphocytes, (**c**) eosinophils, and (**d**) neutrophils in the blood (*n* = 5). * *p* < 0.05, ** *p* < 0.01.

Changes in the proportions of the different types of immune cells in the blood reflect the statuses of the immune systems in the mice following influenza infection. Lymphocytes, neutrophils, monocytes, and eosinophils are all important immune cells involved in combating influenza and disease progression. In the Model group, the blood lymphocyte proportion experienced a notable reduction (Figure 4b), while the proportions of neutrophils, monocytes, and eosinophils were significantly increased (Figure 4a,c,d). The oral administration of the mixed probiotics significantly reduced the proportion of neutrophils in the blood.

### 3.3. Impact of Mixed Probiotics Administration on the Gut Microbiota of Mice

**Figure 5:**
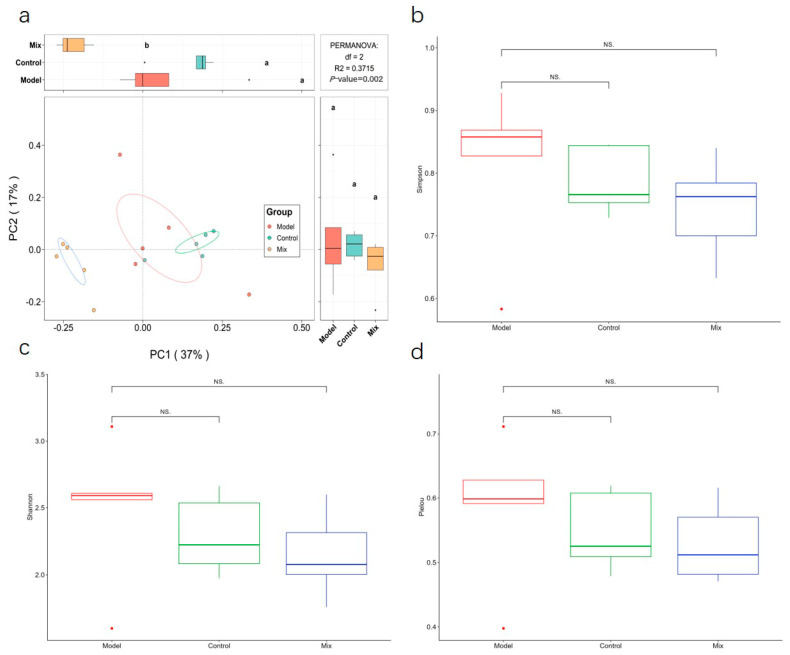
Analysis of the intestinal flora diversity: (**a**) beta diversity analysis, (**b**) Simpson index, (**c**) Shannon index, and (**d**) Pielou index. *n* = 5. Different letters indicate significant differences (*p* < 0.01).

We performed 16S rRNA gene sequencing on the gut microbiome to investigate how the oral intake of a probiotic mixture influences the intestinal microbiota in mice infected with the H1N1 virus. A beta diversity analysis indicated substantial variations in the gut microbiota composition among the different groups of mice (*p* < 0.01) (Figure 5a). In both the Model and Mix groups, the Shannon, Simpson, and Pielou indices showed no significant changes, indicating that the oral administration of mixed probiotics did not significantly affect the alpha diversity of the gut microbiota (Figure 5b).

**Figure 6:**
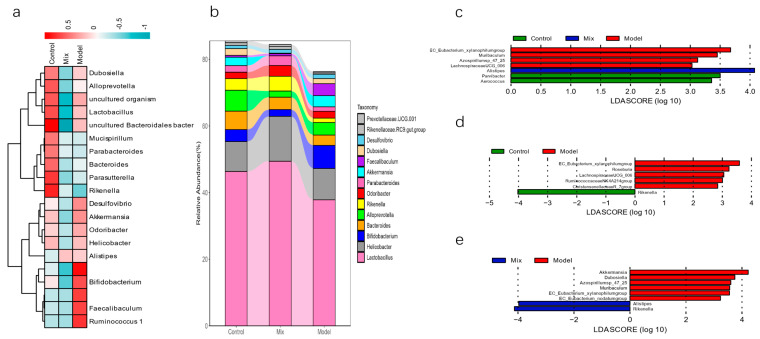
Analysis of the genus-level composition of the mice’s gut microbiomes: (**a**) genetic level categorization of the intestinal microbial community through clustering techniques, (**b**) relative abundance of intestinal microbes at the genus classification level, (**c**) LEfSe analysis of the Control vs. Mix vs. Model groups, (**d**) Control vs. Model groups, and (**e**) Mix vs. Model groups (*n* = 5).

Based on the clustering analysis conducted at the genus level of the gut microbiota, it is evident that the administration of mixed probiotics significantly influenced the relative abundance of gut microbiota genera (Figure 6a). To investigate the precise impacts of the mixed probiotic intervention on the gut microbiota, Linear Discriminant Analysis Effect Size (LEfSe) was applied to the data across all the study groups. The use of mixed probiotics, which alleviated H1N1 infection, significantly increased the abundance of *Alistipes* (Figure 6b). Using the LEfSe analysis to assess the differences in the gut microbiota composition, it was found that, compared to the Control group, the Model group had a significantly increased abundance of *EC_Eubacteriumxylanophilum*, *Roseburia*, *Ruminococcaceae NK4A214*, and *Christensenellaceae R-7*, while the genus *Rikenella* was significantly reduced (Figure 6c). Furthermore, after intervention with mixed probiotics, the abundance of *Alistipes* and *Rikenella* in the mice gut significantly increased, whereas the abundance of *Akkermansia*, *Dubosiella*, *Azospirillum* sp. 47_25, *Muribaculum*, *EC_Eubacterium_xylanophilum*, and *EC_Eubacterium_nodatum* decreased as compared to the Model group (Figure 6d).

### 3.4. Effects of Mixed Probiotics on Mice Intestinal Metabolites

**Figure 7:**
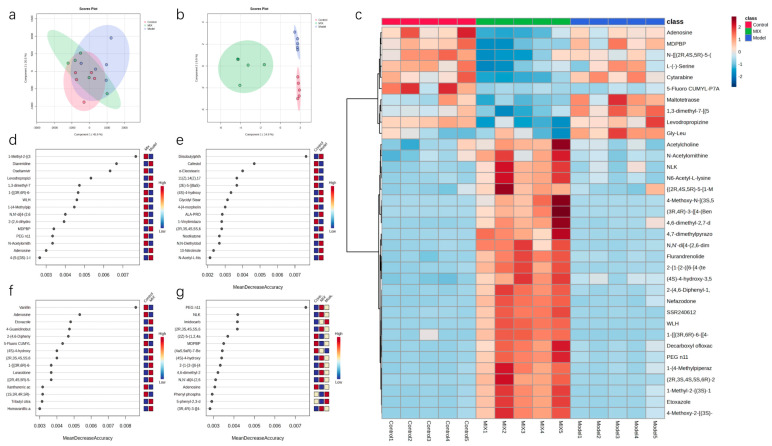
The effect of mixed probiotic treatment on gut metabolites: (**a**) Visualization of the partial least-squares-discriminant analysis (PLS-DA) and (**b**) sparse PLS-DA plots. (**c**) Visualization heatmap of the most significant changes in the intestinal metabolite composition. (**d**–**g**) Random forest analyses of the different groups. Data are presented as the mean ± SEM (*n* = 5).

To investigate the effects of the mixed probiotic intervention on the intestinal metabolites, a non-targeted metabolomic analysis using LC-MS was conducted on fecal samples from the Control, Model, and Mix groups to identify the different metabolites. In total, 602 metabolites were identified. A partial least-squares-discriminant analysis (PLS-DA) and sparse PLS-DA analyses demonstrated significant differences among the Control, Model, and Mix groups (Figure 7a,b). Subsequently, we analyzed the effect of mixed probiotic intervention on the composition of the top 35 metabolites. Following H1N1 viral infection, metabolites such as Gly-Leu, maltotetraose, 1,3-dimethyl-7-[(5-phenyl-1,3,4-oxadiazol-2-yl)methyl]-3,7-dihydro-1H-purine-2,6-dione, and levodropropizine were upregulated, and their abundance became closer to that of the Control group following the mixed probiotic intervention. Six metabolites, including adenosine, were downregulated. Surprisingly, the mixed probiotic intervention enhanced the abundance of 25 metabolites, including acetylcholine, which was in a low abundance state in both the Control and Model groups.

A random forest analysis was employed to pinpoint the metabolites with the greatest significance. Apart from distinguishing between the Control and Model groups, adenosine showed a high discriminatory ability in comparisons across other groups, consistently ranking high and showing a significantly decreased abundance as compared to the Control and Model groups. When comparing the Model and Mix groups and in the combined analysis of all three groups, metabolites such as N,N′-di[4-(2,6-dimethylmorpholino)phenyl]thiourea, MDPBP, PEG n11, and adenosine exhibited high discriminatory power, suggesting that the influence of mixed probiotics on the gut microbiota may be mediated through these metabolites. Hence, these may be key metabolites in the regulation of gut microbiota by both H1N1 influenza virus infection and mixed probiotic intervention.

**Figure 8:**
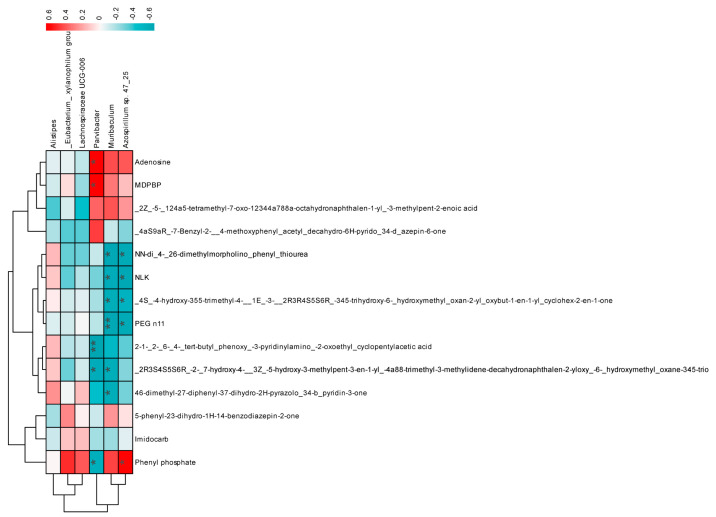
Spearman correlation analysis to examine the relationship between 6 differentially abundant bacteria and 14 metabolites. Data are presented as the mean ± SEM (*n* = 10). The statistical analysis utilized Spearman’s rank correlation coefficient, * *p* < 0.05, ** *p* < 0.01.

We further investigated the connections between the metabolites and the significantly impacted microbial groups. This analysis involved examining the top 14 metabolites and 6 microbial taxa showing differential abundances, followed by a Pearson correlation analysis. *Parvibacter*, *Muribaculum*, and *Azospirillum* sp. 47_25 significantly correlated with most metabolites. Notably, adenosine was significantly positively correlated with *Parvibacter* (Figure 8).

## 4. Discussion

In our study, we demonstrated that the oral administration of mixed probiotics had a beneficial effect in alleviating symptoms in H1N1-infected mice. These effects were primarily reflected in the reduction in body weight loss and the improvement in lung injury following the intervention, consistent with previous reports [15].

Numerous reports have shown that neutrophils and monocytes are highly enriched immune cells following influenza infection and play crucial roles in pathogen recognition and clearance during influenza [15,16]. During infection, respiratory epithelial cells release monocyte chemoattractant protein-1, which attracts monocytes and contributes to the progression of abnormal immune responses [17]. Simultaneously, neutrophils release antimicrobial peptides and structures known as neutrophil extracellular traps, which directly or indirectly impede pathogen proliferation [18]. In our study, we observed an increase in the percentage of neutrophils and monocytes in the blood following influenza infection. After intervention with mixed probiotics, there was a moderate decrease in the percentage of neutrophils and monocytes, indicating immune system engagement and a directional movement of these cells toward the pulmonary region [19]. The dysregulation of glucose metabolism and cytokine storm-induced lymphocyte apoptosis are common phenomena during influenza infection [2,20]. After intervention with mixed probiotics, there was a partial restoration of lymphocyte abundance, suggesting the gradual recovery of the mice toward an uninfected state. Eosinophil levels increase during the initial phases of influenza infection because they participate in viral clearance by releasing inflammatory mediators. Eosinophil levels may gradually decrease in the later stages of infection when the symptoms are alleviated. Mixed probiotics may aid the formation of immune memory, potentially shortening the duration of influenza infections [21]. Considering the close interaction between gut microbiota and the immune system [22], the oral administration of mixed probiotics ultimately reduced the amount of virus present in the lungs of mice, possibly by activating immune cells through the modulation of the gut microbiota to suppress influenza virus replication.

During influenza infection, the host’s immune system releases a large amount of cytokines (such as TNF-α, IL-6, and IL-8) and chemokines, among other inflammatory mediators. These mediators cause vasodilation, capillary leakage, extensive cell adhesion, and the activation of inflammatory cells, leading to tissue damage and functional impairment [23]. The mixed probiotics used in this study exhibited a unique regulatory effect on excessive inflammatory responses during influenza infection. IL-6, TNF-α, and IL-1β are key cytokines involved in early inflammatory responses. IL-6 and IL-1β releases can attract and activate leukocytes, initiating antiviral immune responses. TNF-α enhances vascular permeability and promotes the migration of inflammatory cells [24,25]. In this study, the mice that were infected with influenza and were orally administered the mixed probiotics showed significant reductions in IL-6 and TNF-α levels [26], suggesting a potential alleviation of influenza-induced inflammatory storms. Combining the histopathological evaluation of lung tissues and reduced levels of L-LDH in the BALF, the decreased IL-6 and TNF-α levels effectively controlled lung damage and inflammation. Furthermore, we observed no significant changes in the IL-10 levels, which may have been influenced by the timing of inflammatory cytokine changes during influenza progression and differences in viral strains. Further research is needed to explore these factors at different stages of influenza infection with various strains [27].

Although the influenza virus is primarily transmitted through the respiratory tract, the gut microbiota can also significantly influence the course of influenza infection [28]. First, neither influenza infection nor probiotic intervention significantly modified the alpha diversity of the gut microbiota, indicating a relatively stable richness and evenness level. Compared to the Model group, the mixed probiotics intervention significantly changed the beta diversity of the gut microbiota, suggesting that mixed probiotics can reshape the gut microbiota structure in H1N1-infected mice.

Further analysis of the top 14 species based on relative abundance revealed differences in species richness. Among them, *Lactobacillus* and *Helicobacter* were the predominant species across all three groups of mice. *Rikenella* showed a significantly lower relative abundance in the Model group than in both the Control and Mix groups, consistent with previous reports [29]. It is widely recognized that the introduction of a mixed probiotic containing three strains of *Lactobacillus* typically leads to a notable elevation in the relative prevalence of *Lactobacillus* within the gut microbiota. Lactobacilli are generally recognized for their beneficial effects on the immune system. Many studies have demonstrated that the oral administration of *Lactobacillus* can alleviate influenza infections [30,31]. Based on the LEfSe analysis, the significantly differentially abundant genus in the mixed probiotic group was *Alistipes*, a Gram-negative bacterium belonging to the phylum Bacteroidetes. Although the role of *Alistipes* in influenza infection is not yet clear, its increase in abundance is typically associated with healthier phenotypes in various diseases, such as liver fibrosis, cardiovascular diseases, and cancer immune therapy [32,33]. In studies of colitis, it has been found that the abundance of *Alistipes* is negatively correlated with TNF-α and IL-1β levels [34]. Reports indicate that *Alistipes* produces sulfatides in its metabolites [35]. Moreover, sulfatide drugs can specifically inhibit the macrophage secretion of TNF-α [36], preventing excessive inflammatory responses. Therefore, the control of inflammatory responses by the gut microbiota could be associated with the metabolic activities of *Alistipes*, particularly in the context of sulfatide metabolism.

Based on the analysis of the intestinal metabolites, we identified four compounds that were upregulated in the influenza model. Levodropropizine is a centrally acting antitussive agent that reduces coughing by inhibiting the cough reflex. It is commonly used to treat coughs associated with respiratory diseases such as influenza [37]. Although levodropropizine is currently considered to have no direct immunomodulatory effects on the immune system during influenza, recent clinical studies have shown a positive correlation between blood levels of levodropropizine and eosinophil levels [38]. Considering that levodropropizine can be administered orally, there may be interactions between intestinal metabolites and the blood levels of levodropropizine. These interactions may correlate levodropropizine with the release of inflammatory factors from eosinophils.

Adenosine, a highly discriminative differential metabolite in the gut, has been widely studied for its immunoregulatory function in cancer immunotherapy [39]. Additionally, its metabolite, hypoxanthine [40], has been reported to be upregulated following influenza infection [41], correlating with the pathogenicity of the influenza virus. In this study, mixed probiotics treatment significantly reduced adenosine levels as compared to the other groups, suggesting potential therapeutic benefits for influenza treatment.

## 5. Conclusions

The frequent use of the mixed probiotics of *Lacticaseibacillus rhamnosus* CCFM1279, *Limosilactobacillus reuteri* CCFM1145, and *Lacticaseibacillus casei* CCFM1127 before and after H1N1 infection can help minimize flu symptoms. This is evidenced by a significant reduction in lung damage and pathological inflammation, as well as enhanced immune cell function and systemic immune response. Because this study started administering probiotics before the infection, the gut microbiota had already changed prior to infection. Future research needs to evaluate whether taking probiotics after infection still improves the symptoms of H1N1 infection.

## Figures and Tables

**Table 1:** Primer sequences for the real-time PCR.

Gene	Forward/Reverse	Sequence (5′ to 3′)
*GAPDH*	Forward	AATGGTGAAGGTCGGTGTGAAC
	Reverse	GCCTTGACTGTGCCGTTGAA
*NP*	Forward	GGCACCAAACGGTCTTACGA
	Reverse	TCACCTGATCAACTCCATTACCA
*MxA*	Forward	CCAACTGGAATCCTCCTGGAA
	Reverse	GCCGCACCTTCTCCTCATAG
*Oas1*	Forward	GAAGAGGCTGATGTGTGGCT
	Reverse	TGTCCAGTTCTCTTCTACCTGC

## Data Availability

The original contributions presented in the study are included in the article; further inquiries can be directed to the corresponding authors.
